# A crosstalk of circadian clock and alternative splicing under abiotic stresses in the plants

**DOI:** 10.3389/fpls.2022.976807

**Published:** 2022-10-06

**Authors:** Tao Fan, Mehtab Muhammad Aslam, Jian-Li Zhou, Mo-Xian Chen, Jianhua Zhang, Shenxiu Du, Kai-Lu Zhang, Yun-Sheng Chen

**Affiliations:** ^1^Clinical Laboratory, Shenzhen Children’s Hospital, Shenzhen, China; ^2^Co-Innovation Center for Sustainable Forestry in Southern China & Key Laboratory of National Forestry and Grassland Administration on Subtropical Forest Biodiversity Conservation, College of Biology and the Environment, Nanjing Forestry University, Nanjing, China; ^3^Department of Biology, Hong Kong Baptist University, and State Key Laboratory of Agrobiotechnology, The Chinese University of Hong Kong, Hong Kong, China; ^4^Department of Plant Developmental Biology, Max Planck Institute for Plant Breeding Research, Cologne, Germany

**Keywords:** abiotic stress, circadian clock, plant, signal transduction, splicing regulation

## Abstract

The circadian clock is an internal time-keeping mechanism that synchronizes the physiological adaptation of an organism to its surroundings based on day and night transition in a period of 24 h, suggesting the circadian clock provides fitness by adjusting environmental constrains. The circadian clock is driven by positive and negative elements that regulate transcriptionally and post-transcriptionally. Alternative splicing (AS) is a crucial transcriptional regulator capable of generating large numbers of mRNA transcripts from limited numbers of genes, leading to proteome diversity, which is involved in circadian to deal with abiotic stresses. Over the past decade, AS and circadian control have been suggested to coordinately regulate plant performance under fluctuating environmental conditions. However, only a few reports have reported the regulatory mechanism of this complex crosstalk. Based on the emerging evidence, this review elaborates on the existing links between circadian and AS in response to abiotic stresses, suggesting an uncovered regulatory network among circadian, AS, and abiotic stresses. Therefore, the rhythmically expressed splicing factors and core clock oscillators fill the role of temporal regulators participating in improving plant growth, development, and increasing plant tolerance against abiotic stresses.

## Introduction

### Circadian functional modules

The circadian clock permits an organism temporal coordination of the biological processes at specific times of the day or night, even in the absence of periodicity in the environment. The circadian clock is an endogenous rhythm mechanism in plants which integrates with several external factors to adjust physiological traits of the plants (Covington et al., 2008; Mizuno and Yamashino, 2008; Hsu and Harmer, 2012), providing an advantage to promote plant growth, survival, and adaption.

**Figure 1:**
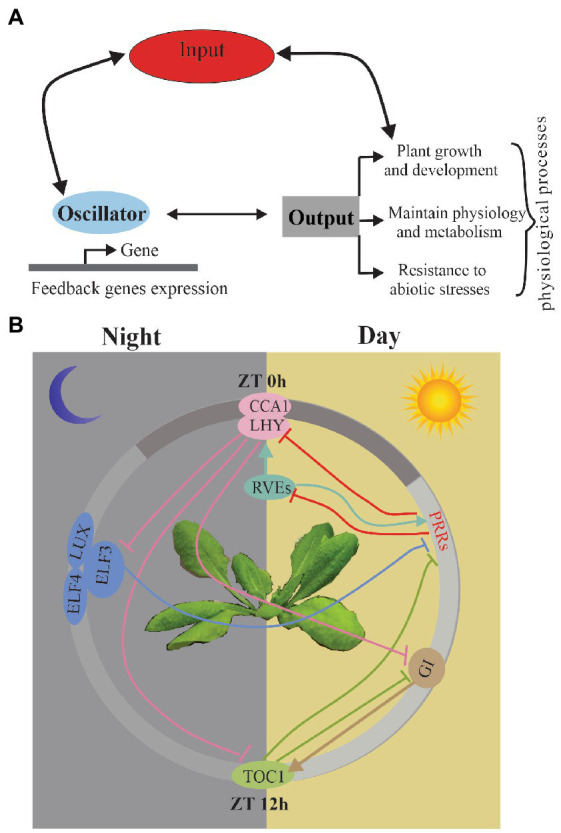
**(A)** Schematic of the circadian clock. A simplified version of the main functional modules of the circadian system; arrows indicate the relationship between modules. **(B)** The current model of *A*. *thaliana* circadian oscillator network with detailed model for transcriptional regulation among the clock components. It shows the relative time of action of each component from left to right in a circadian cycle. The white area indicates subjective day; gray area indicates subjective night. Arrows indicate induction, while perpendicular lines indicate transcriptional suppression.

In the majority of the studies, the circadian clock is predicted to function as a biochemical oscillator with different functional modules, including input, oscillator, and output (Hsu and Harmer, 2014; Greenham and McClung, 2015). Input must be synchronized every day by the diurnal variations to environmental conditions, such as light, temperature, and hormones. (Bordage et al., 2016; Greenwood et al., 2019). Central oscillators are mutually regulated by feedback loops to generate the rhythms in their own expression through transcriptional and post-transcriptional regulation and also drive diverse physiological processes, including growth, flowering time, and stress responses (Figure 1A). Input-oscillator-output is clearly oversimplified as a classical figure view of the circadian system, and now it relies on a much more complex network and can be regulated by other associated life pathways (Harmer, 2009; McClung, 2019).

Based on the importance of clock function, a tremendous amount of progress has been made on understanding the molecular mechanisms responsible for clock entrainment. In the past decades, extensive information on circadian synchronization induced by light has been found through studies on some plants, especially in *Arabidopsis thaliana*. In addition, a large amount of research indicated the involvement of temperature in circadian rhythms (Greenham and McClung, 2015; Philippou et al., 2019). Moreover, other signals such as hormones would entrain plant clock (Atamian and Harmer, 2016).

In *A. thaliana*, a model studying plant circadian clock, the oscillators are regulated transcriptionally and post-transcriptionally through AS, protein–protein interactions, and regulation of protein stability. Components of the circadian oscillator can be described based on the timing of their expression patterns with morning-phased (*CCA1* and *LHY*), daytime-phased (*PRR7*, *PRR9*), afternoon-phased (*RVE4*, *RVE6* and *RVE8*), and evening-phased genes (*PRR5*, *TOC1*, *LUX*, *ELF3*, *ELF4,* and *GI*) (Greenham and McClung, 2015; Philippou et al., 2019). The widespread feedback loops regulate the expression of each gene and their downstream targets all day (Harmer, 2009; Pokhilko et al., 2013; Fogelmark and Troein, 2014; Hsu and Harmer, 2014; McClung, 2014) (Figure 1B). In the morning phase, a motif exists in the *TOC1* promoter named the evening element (EE), CIRCADIAN CLOCK-ASSOCIATED 1 (*CCA1*) and LATE ELONGATED HYPOCOTYL (*LHY*) repress the expression of an evening-phased component TIMING OF CAB EXPRESSION 1 (*TOC1*) (Endo et al., 2014). Recently, *CCA1* and *LHY* have also been shown to repress several other evening genes, including GIGANTEA (*GI*), LUX ARRHYTHMO (*LUX*), EARLY FLOWERING 3 (*ELF3*), and *ELF4* (McClung, 2011; Endo et al., 2014; Nolte and Staiger, 2015). *CA1* and *LHY* repress evening genes depending on DEETIOLATED1 (*DET1*), a key repressor in photo morphogenesis (Romanowski and Yanovsky, 2015). In the day phase, *PRRs* (*PRR9*, *PRR7* and *PRR5*) could repress the expression of the morning genes *CCA1* and *LHY* (James et al., 2012). Recently, the PRRs have been shown to also repress expression of REVEILLE8 (*RVE8*) (McClung, 2014). In the afternoon phase, *RVE8* is associated with *PRR5* and *TOC1* promoters (Salome et al., 2002; Harmer, 2009). *RVE4* and *RVE6*, two close homologs of *RVE8*, also play the same roles as *RVE8* in the circadian system (Harmer, 2009). In the evening phase, similar to *TOC1* homologs *PPRs*, *TOC1* represses *CCA1* and *LHY* expression (Pokhilko et al., 2013; de Montaigu et al., 2015). Moreover, *LUX*, *ELF3*, and *ELF4* represses the expression of the day-phased clock gene *PRR9* (Hsu and Harmer, 2014). *GI* have been verified to regulate the *TOC1* expression.

The central oscillator can generate rhythmic outputs, including growth and development, stress, and metabolism (Greenham and McClung, 2015; Malapeira et al., 2015; Atamian and Harmer, 2016). The circadian clock generates rhythms in plant growth and development processes such as hypocotyl growth, hormone signaling, germination, leaf and root growth, architecture, and flowering time (de Montaigu et al., 2010; Stitt and Zeeman, 2012; Kinmonth-Schultz et al., 2013; Johansson and Staiger, 2015). Moreover, plant metabolism is an important output, influencing starch metabolism, photosynthesis, fitness, biomass, metabolite profiling second messengers, micronutrients, and mineral homeostasis (Haydon et al., 2013; Malapeira et al., 2015). In addition, under circadian clock control, cold, drought, salinity, and reactive oxygen species (ROS) could also respond (Legnaioli et al., 2009; Grundy et al., 2015).

### Abiotic stresses and plant biology

**Figure 2:**
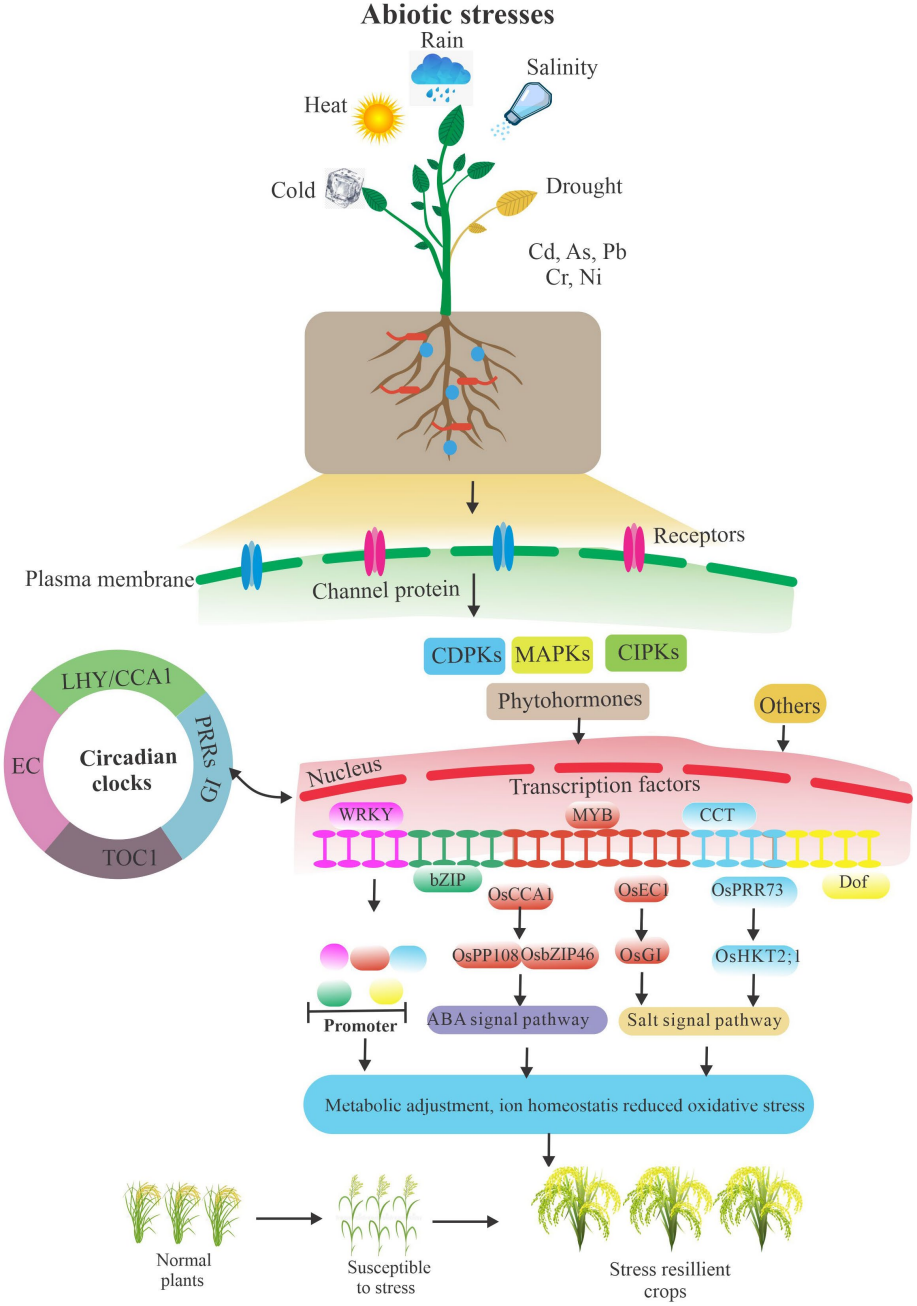
A simplified schematic of abiotic stresses and plant biology. Plants resist abiotic stresses by interacting with signaling pathways, circadian clocks, hormones, crucial genes transcriptional and post-transcriptional regulation, and other ways under environmental challenges. Others included endophytic fungi, a self-antioxidant defense system, proline accumulation, and so on. The arrows indicate the relationship between modules.

Abiotic stresses are the major environmental constraints that affect plant growth and productivity due to rapid fluctuations in ambient conditions. To adapt to abiotic stress challenges, plants can initiate appropriate molecular, cellular, and physiological adjustments to survive and reproduce (Figure 2). Plants transmit the stress signals within cells as well as between cells and tissues, and activate stress-related genes to respond to stress signals through transcriptional and post-transcriptional regulation, particularly splicing factor mediated pre-RNA splicing and DNA methylation (Bertolini et al., 2018; Zhang et al., 2018; Huertas et al., 2019; Chen et al., 2020). Moreover, hormones play a pivotal role in plant development and signaling networks regulating plant responses to a wide range of environmental conditions. Plant hormones fasten on the significant link between different hormones in response to stress, such as ABI5 in relation to other phytohormones involved in the abiotic stress responses, apocarotenoids which induce other phytohormones critical for plant growth, development, and stress response, and gibberellins which act as a promoter in primary root development (Skubacz et al., 2016; Verma et al., 2016; Felemban et al., 2019; Lopez-Ruiz et al., 2020). Indeed, the circadian clock regulates environmental stresses and plant biological processes, particularly the major circadian clock genes, such as *PRR5* and *PRR7* in *A. thaliana*, and *LHY* in *A. thaliana* and *Glycine max* (Kolmos et al., 2014; Nakamichi et al., 2016; Yoo et al., 2020; Wang K. et al., 2012). Moreover, *OsPRR73*, a circadian component, may confer salt tolerance by recruiting *HDAC10* to repress *OsHKT2;1*, thus decreasing cellular Na(+) accumulation (Wei et al., 2021). In addition, the rice evening complex, composed of *OsELF4a*, *OsELF3-1,* and *OsLUX*, could regulate heading date and salt tolerance. Interestingly, in long day conditions, *GI* mutant *osgi-101* showed salt tolerance and exhibits early heading phenotype. Compelling evidence shows that *OsEC1* represses *OsGI* and thus links the circadian clock with salt tolerance (Wang X. et al., 2012). Moreover, DNA affinity purification sequencing (DAP-seq) coupled with transcriptome analysis indicates a direct transcriptional target of *OsCCA1*, substantially enriched in ABA signaling pathway. Rice *CCA1* could transcriptionally regulate ABA signaling to confer tolerance to multiple abiotic stresses by a direct association of *OsPP108* and *OsbZIP46* promoters (Wei et al., 2022).

Plants adapt to various environmental stresses by endophytic fungi, a self-antioxidant defense system, proline accumulation, and so on (Khan et al., 2015; Wani et al., 2019; Hasanuzzaman et al., 2020). Interestingly, transcriptional regulation also plays an important role in plant biological processes from plant development to stress responses (Thatcher et al., 2016; Shang et al., 2017; Calixto et al., 2018; Szakonyi and Duque, 2018; Nimeth et al., 2020). In natural and agricultural backgrounds, plants also constantly suffer from environmental conditions. Stress mediates plant SR protein genes’ expression changing at the transcriptional and transcriptomic level (Zhang et al., 2020). Data certified that drought induced large developmental splicing changes in leaf and ear but relatively few in tassel.

To date, several studies documented the crosstalk between the circadian clock and AS, including splicing the clock to maintain and entrain circadian rhythms in *Drosophila melanogaster* (Shakhmantsir and Sehgal, 2019), chromatin remodeling, and AS of the *A. thaliana* circadian clock (Henriques and Mas, 2013). In this study, we paid more attention to the links between circadian and AS of plant life activities responsive to abiotic stresses, making it different from the published articles.

## An emerging link between circadian clock and AS under abiotic stresses

**Figure 3:**
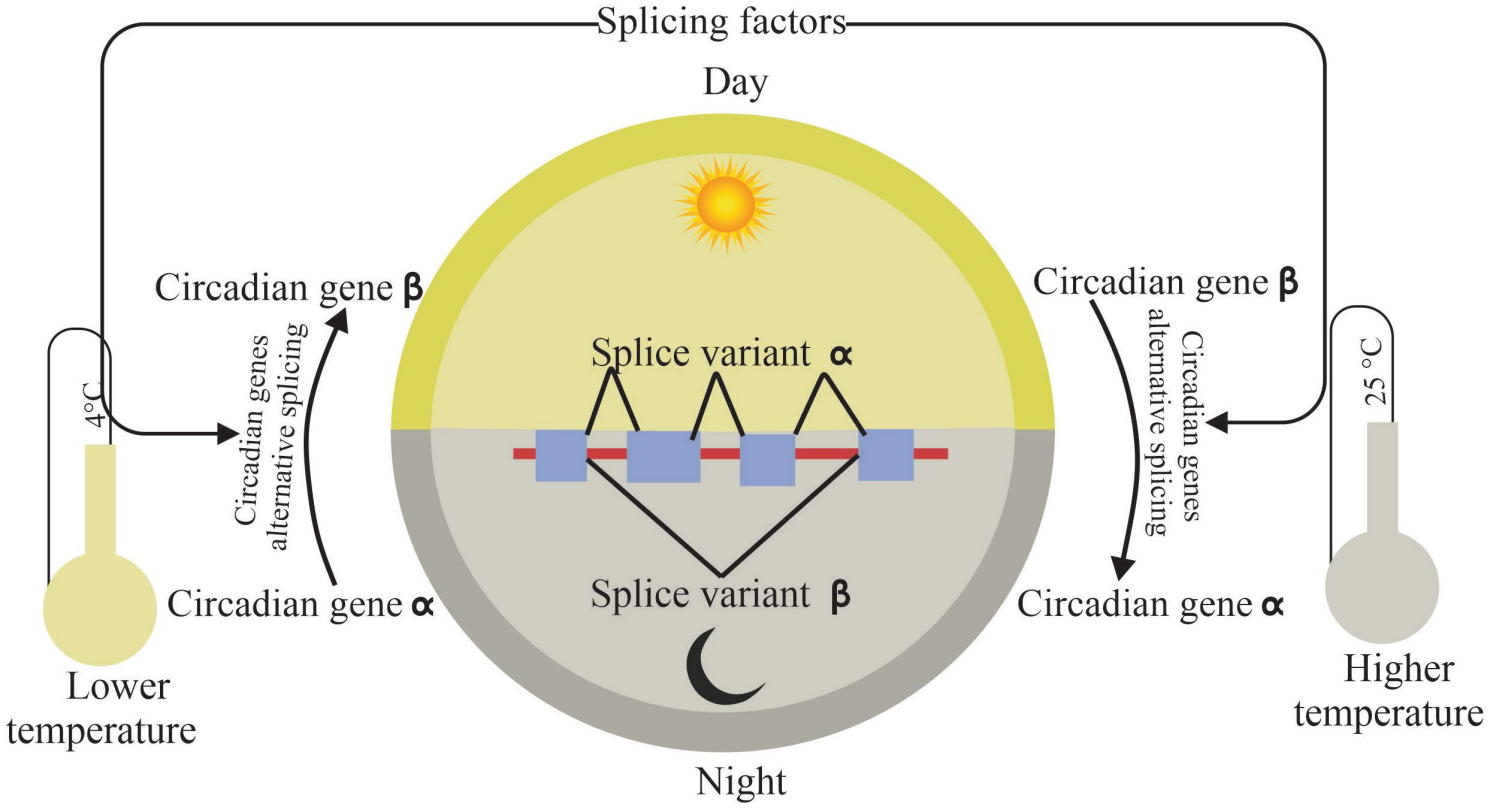
Connection of circadian and alternative splicing. Splicing factors affect circadian rhythm by self and core circadian genes splicing under temperature stress, while circadian-mediated alternative splicing according to rhythmic temperature changes in day and night. The arrows indicate the relationship between modules.

Research into what regulates circadian and AS offered more details about the fragile crosslink under environmental stresses, especially temperature changes (Figure 3).

### Splicing factors affect circadian rhythm by self and core circadian genes splicing under abiotic stresses

As a post-translational controller, AS plays a prominent role in the circadian clock, especially in spliceosome. SPLICEOSOMA L TIMEKEEPER LOCUS1 (*STIPL1*), a component of *A. thaliana* spliceosome, could affect the circadian period. There exists two STIPL splicing transcripts in *A. thaliana*. Interestingly, mutation of another *STIPL2* does not cause splicing defects or changes in circadian rhythms in *A. thaliana*, diverging from *STIPL1* (Bertoni, 2012), which may reveal the function of splicing factors in AS. SNW/Ski-interacting protein (*SKIP*, splicing factor), a component of the spliceosome, physically associates with the spliceosomal splicing factor Ser/Arg-rich protein45 and interacts with the pre-mRNA of clock genes, such as *PRR9* and *PRR7*, involved in AS and mRNA maturation. Genome-wide investigations show that SKIP participate in regulating AS of several genes, possibly *via* modulating recognition or cleavage of 5′ and 3′ splice donor and acceptor sites. Therefore, *SKIP* could link AS and the circadian clock as post-transcriptional regulation in *A. thaliana* (Wang et al., 2012). Moreover, growing evidence suggests that some *LSM* genes encode core components of the spliceosome U6 complex which regulate circadian rhythms in both plants and mammals. The expression and AS of some core clock genes were different in *A. thaliana lsm5* mutants. Later, expression analysis of either a weak *lsm5* or a strong *lsm4* mutant allele in *A. thaliana* displayed larger effects on AS than on constitutive splicing, including circadian clock genes. Notably, RNA-seq reveals large splicing defects were not observed in most of the introns evaluated in the strong *lsm4* mutant allele, especially circadian clock genes. These findings support the idea that some *LSM* genes influence core clock genes AS, particularly intron retention events (Perez-Santangelo et al., 2014). Meanwhile, the spliceosome assembly factor *GEMIN2* controls the AS of several clock genes and deadens caused by temperature on the circadian period in *A. thaliana* (Schlaen et al., 2015). Recently it has been discovered that *PRMT5*, a protein arginine methyl transferase, adds a new loop within the circadian clock of the *A. thaliana* by regulating AS of main clock genes. Meanwhile, investigations showed that *PRMT5* has a role in the regulation of AS and the circadian network in *D. melanogaster* (Petrillo et al., 2011). Thus, these major spliceosomes can reveal several abiotic stress responses, and a link between AS and circadian rhythm changes.

Interestingly, the circadian clock could deal with the environmental changes by AS. Temperature variation can cause extensive dynamic changes in AS of clock genes and alternatively spliced transcripts. Temperature-associated AS is an additional loop to regulate the plant circadian clock (James et al., 2012). CIRCADIAN CLOCK-ASSOCIATED1 (*CCA1*), a core clock component, can be self-regulated by a splice variant *CCA1β* which is inhibited by low temperatures, while *CCA1β* suppresses the activities of functional *CCA1α* and LATE ELONGATED HYPOCOTYL (*LHY*) transcription factors by forming nonfunctional *CCA1α-CCA1β* and *LHY-CCA1β* heterodimers in *A. thaliana* (Park et al., 2012). While in control condition, *CCA1α* actively expresses without binging *CCA1β*. Moreover, frequency of AS, *FRQ*, a circadian clock gene, exhibits a robust circadian rhythm and regulates the response of the circadian clock to temperature changes. *l-FRQ* has a normal expression in condition temperatures. But the amount of *l-FRQ* increases significantly as temperatures rise, whereas *s-FRQ* levels increase while temperature is low. This leads to a variation in the *l-FRQ* to *s-FRQ* ratio as a function of temperature (Diernfellner et al., 2005, 2007). A later study investigated how the major clock genes, such as TIMING OF CAB EXPRESSION 1 (*TOC1*) and EARLY FLOWERING 3 (*ELF3*), undergo extensive AS under all sorts of environmental conditions, indicating AS creates a linkage between the circadian clock and environmental stress adaptation in plants (Kwon et al., 2014). Compared to normal conditions, *TOC1* and *ELF3* increase considerably in intron retention events under cold conditions. In recent years, new technology tools such as global circadian RNA-seq event centered, a splicing analysis tool, offer a new approach for study between the circadian clock and splicing events. Interestingly, expression of AS events of the circadian clock vary corresponding with the season and temperature in sugarcane (Dantas et al., 2019). That indicated plants deal with abiotic stress by major gene rhythmic expression which is regulated by AS.

### Circadian-mediated AS

Much evidence indicated some genes participating in abiotic stress responses, especially temperature stress, could undergo AS according to circadian rhythm changes. AtGRP7, a protein which is mediated by the circadian clock, increases stress tolerance under cold conditions and through this undergoes circadian oscillations. The protein can also autoregulate its expression by binding to its own pre-mRNA to influence its own AS (Staiger et al., 2003). Moreover, in *D. melanogaster*, splicing of an intron at the 3′ untranslated region of the period (per) mRNA is enhanced at cold temperatures, which reveals that daily fluctuations in the splicing of intron is regulated in a manner that depends on the photoperiod and temperature (Majercak et al., 2004). In addition, in plants, daily and circadian oscillated gene PROTEIN ARGININE METHYL TRANSFERASE 5 *(PRMT5*), involved in vernalization and carrying methyl groups to arginine residues present in histones and Sm spliceosome proteins, could link the circadian clock to controlling AS. Mutant *Atprmt5* impairs several circadian rhythms and phenotypes resulting from AS of the core-clock gene *PRR9*. Further studies show that *PRMT5* participates in the regulation of many pre-messenger-RNA splicing events, probably by modulating 5′-splice-site recognition, which indicates *PRMT5* could link the circadian clock and AS to help organisms to synchronize physiological progresses to deal with daily changes in environmental conditions (Sanchez et al., 2010). There are few papers that focus on the changes of expression of key regulators of AS regulated by a circadian clock under both control and abiotic stress conditions, which provides us a novel viewpoint to explore in the future.

## Conclusions and future perspectives

Numerous scholarly articles focus on AS, the circadian clock, and abiotic stress, but very little evidence pointed out how to deal with abiotic stresses *via* circadian-mediated AS in plants. The circadian clock regulates AS in a tissue-dependent manner and concurrent with circadian transcript abundance (McGlincy et al., 2012), found in different tissues and circadian transcript. One could hypothesize that the genes undergo regular splicing variant in tissues and development stages. Once the environment changes, the major splicing variant expressed more to respond to the particular stress. Recent research indicates the clock is a temporal regulator of AS (Genov et al., 2019), which may give us a possible explanation about temporal diversification of the proteome. More evidence indicates that AS events corresponding with circadian are widespread across mammalian tissues and might conduce to a temporal diversification of the proteome (El-Athman and Relogio, 2018). However, it remains to be determined which genes are the temporal regulator in plant life progress. The rhythmically expressed splicing factors and core clock oscillators fill the role of temporal regulators to participate in plant growth, development, and dealing with abiotic stresses. Those data present global circadian RNA-seq event, using a splicing analysis tool to shed light on the possible relationship between AS, circadian, and abiotic stresses.

Moreover, phytohormones play a critical role in plant adaptation to environmental stresses; the circadian clock could respond to these stresses through AS events (Figure 2). However, only a few studies have reported on how this process is mediated. A board range of studies investigated temperature variations, while only a few were conducted on drought and salinity stress. The abscisic acid (ABA), known as a plant stress hormone, plays a major role in abiotic stress responses, particularly under drought and salt stress (Zhang et al., 2006; Ma and Qin, 2014). Interestingly, the circadian clock regulates ABA signaling by circadian oscillator. There are bidirectional interactions between the circadian oscillator TIMING OF CAB2 EXPRESSION1 (*TOC1*) and ABA signaling (Legnaioli et al., 2009). ABA-inducible R2R3-type MYB transcription factor, *MYB96*, binds directly to the *TOC1* promoter to activate its expression; *TOC1* in turn regulates *MYB96* expression possibly *via CCA1*. The whole complex *CCA1-MYB96-TOC1* circuit connects circadian and ABA signaling to address abiotic stress (Lee et al., 2016). LATE ELONGATED HYPOCOTYL (*LHY*), a circadian oscillator, binds directly on the promoters of genes in ABA signal pathway, connecting circadian regulation with drought and salt stress tolerance through ABA signaling (Belbin and Dodd, 2018). More evidence indicates that AS regulates stress responses largely by targeting the ABA pathway. For instance, *A. thaliana* seedlings treated with ABA demonstrated varied conventional AS isoforms expression and increased non-conventional AS events number (Zhu et al., 2017). In agreement, plant mutant defective in splicing factors are severely impaired in their response to abiotic stress, such as *STA1* (Lee et al., 2006), *LSM4* (Zhang et al., 2011), and *SKIP* (Feng et al., 2015). These findings provide us a possible hypothesis: splicing factors may target circadian oscillator genes splicing or splicing factors *via* self-splicing to joint circadian oscillator genes to link ABA signaling pathway and respond to abiotic stress.

Auxin, another plant hormone, plays key roles in plant development and responses to environmental cues, and there is a new crosslink of the clock and auxin. The circadian clock core gene REVEILLE1 (*RVE1*) could regulate the expression of the auxin biosynthetic gene YUCCA8 (*YUC8*), suggesting a mechanism for coordinating plant growth with rhythmic changes in the variable environment (Rawat et al., 2009). CIRCADIAN CLOCK-ASSOCIATED1 (*CCA1*) plays an essential role in gating auxin response (Xue et al., 2020). Interestingly, splice variant *CCA1β* inhibited by low temperature could suppress the function of another splice variant *CCA1α*, which exists as a new net between circadian and AS response to abiotic stresses through hormonal signaling pathways.

## Author contributions

Y-SC and K-LZ: conceptualization. TF, MA, J-LZ, and M-XC: writing original draft preparation. TF, M-XC, J-LZ, SD, and Y-SC: writing review and editing. Y-SC: funding. All authors have read and agreed to the published version of the manuscript.

## Funding

This work was supported by the Science Technology and Innovation Committee of Shenzhen (2021N062-JCYJ20210324115408023), the Natural Science Foundation of Jiangsu Province (SBK2020042924), the National Natural Science Foundation of China (32001452), and the Hong Kong Research Grant Council (AoE/M-403/16, GRF14160516, 12100318 and 12103220).

## Conflict of interest

The authors declare that the research was conducted in the absence of any commercial or financial relationships that could be construed as a potential conflict of interest.

## Publisher’s note

All claims expressed in this article are solely those of the authors and do not necessarily represent those of their affiliated organizations, or those of the publisher, the editors and the reviewers. Any product that may be evaluated in this article, or claim that may be made by its manufacturer, is not guaranteed or endorsed by the publisher.
